# Supplementary material for: Cystic fibrosis risk variants confer protection against inflammatory bowel disease
Source: Cell Genom. 2025 Dec 1;6(2):101071. doi: 10.1016/j.xgen.2025.101071 (PMC12903406; doi:10.1016/j.xgen.2025.101071)
Supplement: Document S1. Figures S1–S4 and Tables S2, S3, S5, and S6 [file mmc1.pdf]

Cell Genomics, Volume *6*

Supplemental information

Cystic fibrosis risk variants confer protection against inflammatory bowel disease

Mingrui Yu, Qian Zhang, Kai Yuan, Aleksejs Sazonovs, Christine R. Stevens, Laura Fachal, International Inflammatory Bowel Disease Genetics Consortium Sequencing Group,, Christopher A. Lamb, Carl A. Anderson, Mark J. Daly, and Hailiang Huang

Cystic fibrosis risk variants confer protection against inflammatory bowel disease

Mingrui Yu[1,2,3], Qian Zhang[4,5,6], Kai Yuan[1,2,3,6], Aleksejs Sazonovs[4], Christine R. Stevens[1,2,3,6], Laura Fachal[4,5,6], International Inflammatory Bowel Disease Genetics Consortium Sequencing Group[+], Christopher A. Lamb[5,6,7,8], Carl A. Anderson[4,5,6,*], Mark J. Daly[1,2,3,6,9,10*], Hailiang Huang[1,2,3,6,10,11*]

[1]Program in Medical and Population Genetics, The Broad Institute of MIT and Harvard, Cambridge, MA, USA
[2]Stanley Center for Psychiatric Research, the Broad Institute of MIT and Harvard, Cambridge, MA, USA
[3]Analytic and Translational Genetics Unit, Department of Medicine, Massachusetts General Hospital, Boston, MA, USA
[4]Genomics of Inflammation and Immunity Group, Human Genetics Programme, Wellcome Sanger Institute, Wellcome Genome Campus, Hinxton, Cambridgeshire, UK;
[5]UK Inflammatory Bowel Disease Genetics Consortium
[6]International Inflammatory Bowel Disease Genetics Consortium
[7]The Royal Victoria Infirmary, Newcastle upon Tyne NE1 4LP, UK
[8]NIHR IBD BioResource
[9]Institute for Molecular Medicine Finland, FIMM, HiLIFE, University of Helsinki, Helsinki, Finland
[10]NIDDK Inflammatory Bowel Disease Genetics Consortium

[11]Lead contact
[+]A list of members of the consortium can be found in Table S7.
*Correspondence: ca3@sanger.ac.uk (C.A.A.), mjdaly@broadinstitute.org (M.J.D.), hhuang@broadinstitute.org (H.H.)

Figure S1: Data quality control workflow.
Figure S2: Impact of undetected CF patients in the Broad EUR control.
Figure S3: Variant quality control metrics.
Figure S4: Admixture proportions in CF-risk variant carriers and non-carriers.

Table S2: Number of potential CF patients removed from each study in EUR ancestry
Table S3: Association result of deltaF508 after excluding potential CF patients
Table S5: CF-risk variant burden test in CD and UC
Table S6: Burden test for EAS and AFR.AMR groups

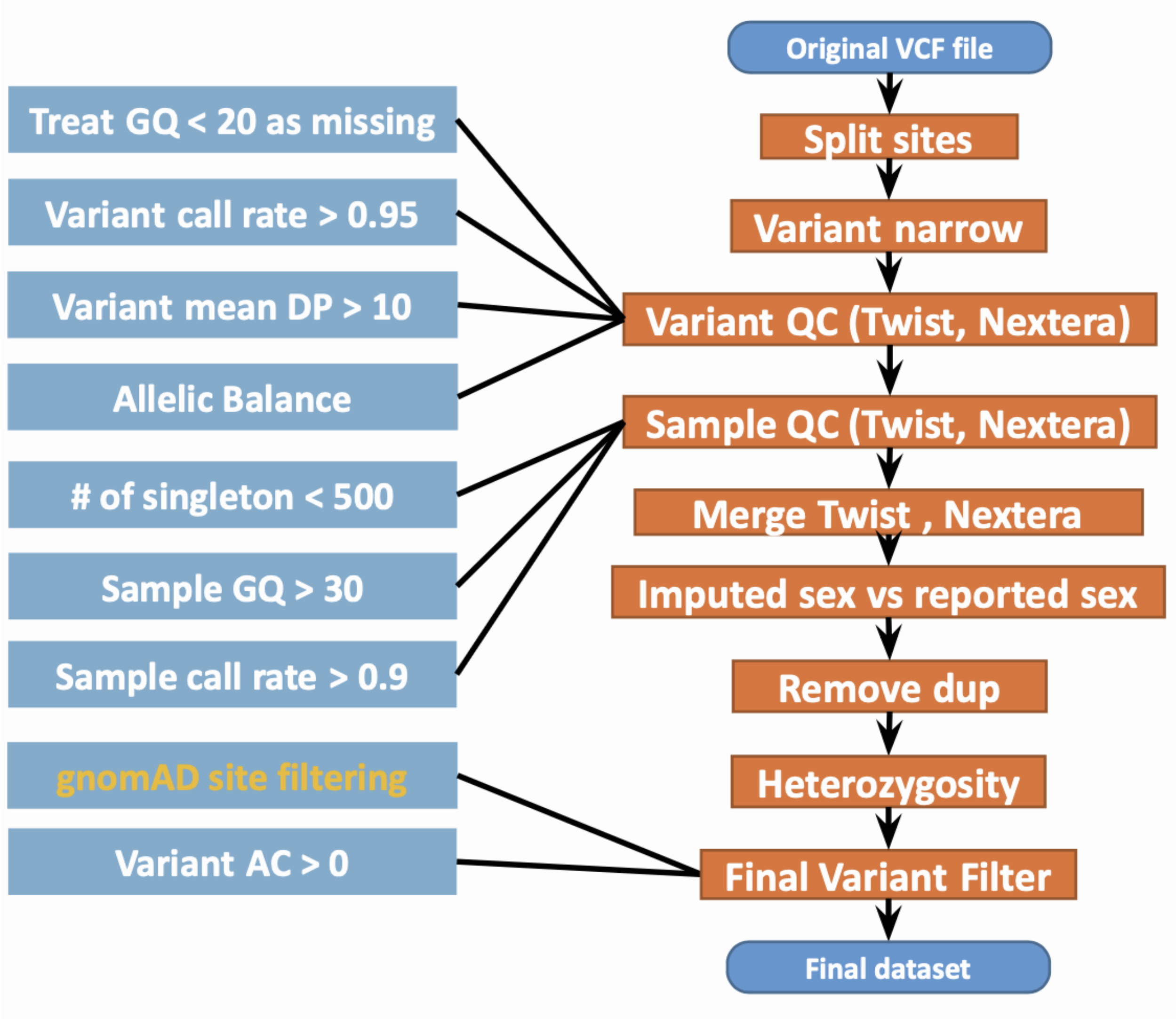

Figure S1: Data quality control workflow, related to STAR Methods. Quality control steps performed on variants and subjects on the Broad sequencing dataset. Details and specific parameters are described in Methods.

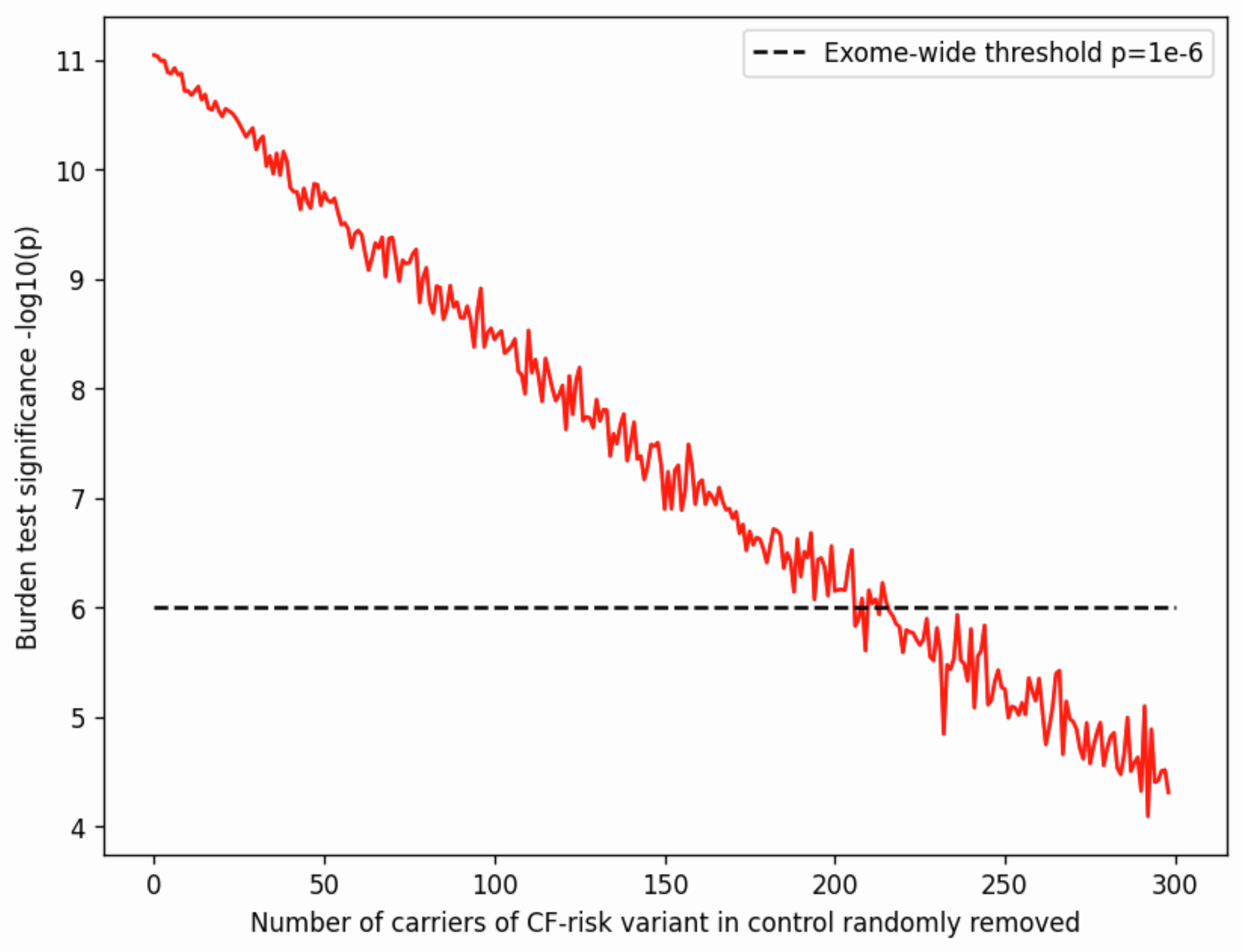

Figure S2: Impact of undetected CF patients in the Broad EUR control, related to Tables 2 and 3. Out of 3290 control carriers of CF-risk variants in Broad EUR, a subset was randomly selected and removed as "undetected CF patients" in simulations. CF-risk variant burden tests, including deltaF508, were performed. Burden test significance and number of carriers removed for each time were plotted to gauge the sensitivity of association significance to the number of undetected CF patients. 114 potential CF patients in Broad controls were previously identified and removed (Table S2). An additional ~200 CF-risk variant carriers in control need to be removed for the burden test significance to drop below the exome-wide significance threshold.

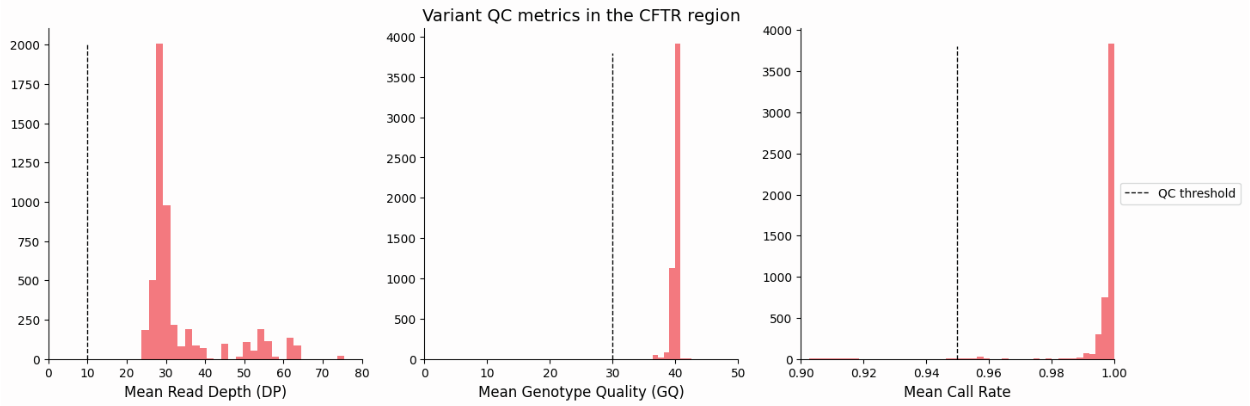

Figure S3: Variant quality control metrics, related to STAR Methods. Distributions for variant-level QC metrics (mean read depth, mean genotype quality, and mean call rate) for all variant sites within the CFTR locus (GRCh38 chr7:117480020-117668665) are plotted. Dashed lines indicate the chosen threshold for variant filtering in QC.

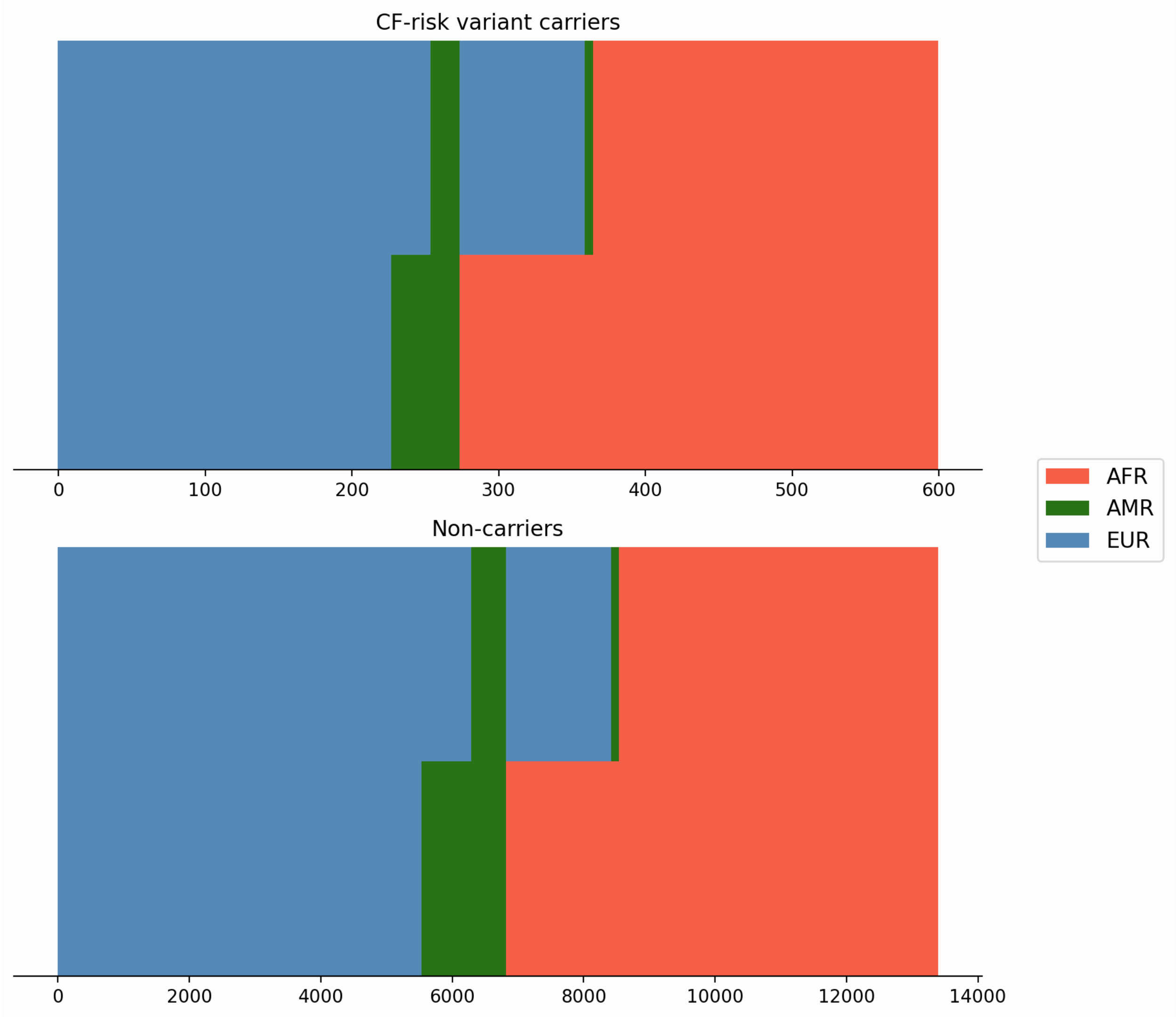

Figure S4: Admixture proportions in CF-risk variant carriers and non-carriers, related to STAR Methods. Local ancestry inference (Methods) was performed for each individual in the *CFTR* locus (GRCh38 chr7:117480020-117668665). Each haplotype was assigned to one of three ancestries: AFR, AMR, and EUR. Each vertical bar represents one individual, where the colors represent the ancestries from which the haplotypes came. Individuals are sorted by their ancestry admixture for plotting.

Study	Number of homozygous carriers removed (case)	Number of homozygous carriers removed (control)	Number of potential compound homozygous carriers removed (case)	Number of potential compound homozygous carriers removed (control)
Broad WES	2	46	19	68
Sanger WES	1	1	12	78
Sanger WGS	1	0	6	24

Table S2: Number of potential CF patients removed from each study in EUR ancestry, related to Table 2. Homozygous and compound homozygous carriers of CF-risk variants are treated as potential CF patients and removed from analysis.

Study	Subtype	MAF(case)	MAF(control)	p-value	OR	OR (95% CI)
Broad WES	CD	0.00967	0.0127	5.96E-06	0.76	0.67 - 0.85
	UC	0.00962	0.0127	3.89E-03	0.83	0.73 - 0.94
	IBD	0.00965	0.0127	2.14E-07	0.78	0.70 - 0.85
Sanger WES	CD	0.01330	0.015986	2.34E-02	0.86	0.76 - 0.98
	UC	0.01409	0.015986	2.61E-01	0.94	0.84 - 1.05
	IBD	0.01362	0.015986	1.46E-02	0.90	0.82 - 0.98
Sanger WGS	CD/IBD*	0.01128	0.015165	1.76E-02	0.75	0.59 - 0.95
**Meta-analysis**	**CD**	**-**	**-**	**8.84E-08**	**0.80**	**0.73 - 0.86**
	**UC**	**-**	**-**	**5.40E-03**	**0.89**	**0.81 - 0.96**
	**IBD**	**-**	**-**	**1.16E-08**	**0.84**	**0.78 - 0.88**

Table S3: Association result of deltaF508 after excluding potential CF patients, related to Table 2. MAF: minor allele frequency; OR, SE: odds ratio and standard error from the logistic mixed model; 95% CI: 95% confidence interval of OR. *Sanger WGS only included CD patients.

Study	Disease	Clinical annotation	# of Variants	CAF	OR	p-value	OR (95% CI)
Broad WES	CD	CF-risk	132	0.012	0.852	3.00E-03	0.762 - 0.953
Broad WES	UC	CF-risk	130	0.012	0.756	1.40E-05	0.661 - 0.864
Sanger WES	CD	CF-risk	129	0.011	0.932	3.83E-01	0.810 - 1.074
Sanger WES	UC	CF-risk	131	0.011	0.844	1.49E-02	0.734 - 0.970
Sanger WGS	CD/IBD*	CF-risk	56	0.023	0.852	0.07	0.717 - 1.013
**Meta-analysis**	**CD**	**CF-risk**	**-**	**-**	0.878	**8.8E-04**	**0.813 - 0.948**
**Meta-analysis**	**UC (no Sanger WGS)**	**CF-risk**	**-**	**-**	0.795	**3.6E-06**	**0.722 - 0.875**

Table S5: CF-risk variant burden test in CD and UC, related to Table 3. CAF: composite allele frequency, defined as the frequency of observing carriers of at least one variant of interest in the study. *Sanger WGS only included CD patients.

Ancestry	IBD Cases	Controls	N_eff	# of CF-risk variants	CAF	OR	p-value	OR (95% CI)
AFR.AMR	4421	9976	12253	76	0.0217	0.793	0.023	0.647 - 0.971
EAS	1624	1462	3079	7	0.0044	0.164	0.011	0.032 - 0.837
EUR (Broad discovery)	38558	66945	97865	142	0.0231	0.791	9.01E-12	0.739 - 0.847

Table S6: Burden test for EAS and AFR.AMR groups, related to Tables 2 and 3. Burden tests of all CF-risk variants, including deltaF508, were performed for AFR.AMR and EAS ancestral groups.
